# Discrepancy between objective and subjective measurements of sleep quality: the role of panic-agoraphobic spectrum

**DOI:** 10.1192/j.eurpsy.2023.605

**Published:** 2023-07-19

**Authors:** F. Cruz Sanabria, M. Violi, L. Massoni, C. Bonelli, D. Gravina, S. Bruno, U. Faraguna, L. Dell’Osso, C. Carmassi

**Affiliations:** ^1^Department of Translational Research and of New Surgical and Medical Technologies; ^2^Department of Clinical and Experimental Medicine, University of Pisa, Pisa, Italy

## Abstract

**Introduction:**

There is evidence that anxiety and depressive symptoms may lead individuals to under-estimate their own sleep quality, particularly among younger subjects (aged <45 yrs).

**Objectives:**

The aim of this study was to investigate the discrepancy between objective and subjective measurements of sleep quality in a sample of healthy control subjects (HCs) with no Axis I mental disorders, and the possible impact of panic-agoraphobic spectrum symptoms.

**Methods:**

A total of 117 HCs (65 males and 97 females; Age: 35.3±14yrs) were evaluated by the: Panic Agoraphobic Spectrum-Self Report (PAS-SR), to investigate panic spectrum; the Pittsburgh Sleep Quality Index (PSQI) and actigraphy, respectively for the subjective and the objective sleep efficiency (SE) measures. Groups were divided according to the congruence between SE-actigraphic vs SE-PSQI (“Accurate”, “Underestimate”, “Overestimate”), establishing as a threshold an SE>85% as a measure of good SE. Regression analyses were conducted to assess the association between PAS-SR domains and the discrepancy between objective and subjective measurements, controlling confounding factors such as age, gender and BMI

**Results:**

Since our data showed that a low sleep quality was associated with a greater age and that higher PAS-SR scores were associated with younger age, we used a sub-sample of 117 participants with age <45 years and comparing the 3 groups of subjects created on the basis of the discrepancy: Accurate, N = 74 (63.2 %), “Overestimate group”, N= 23 (19.7 %), “Underestimate group”: N=20 (17.1 %), we found a statistically significant difference among groups in the PAS.SR separation anxiety domain (p value=0.032), with a multinomial regression model confirming this domain contributed significantly to the differentiation between the three groups with higher symptoms being associated with a higher probability of belonging to the “underestimate” group.

**Conclusions:**

Our results suggest that the discrepancy between objective and subjective sleep efficiency measurements in HCs could be affected by panic spectrum symptoms, particularly separation anxiety.

**Disclosure of Interest:**

None Declared

